# Percolated Network of Mixed Nanoparticles with Different Sizes in Polymer Nanocomposites: A Coarse-Grained Molecular Dynamics Simulation

**DOI:** 10.3390/ma14123301

**Published:** 2021-06-15

**Authors:** Xiuying Zhao, Yun Nie, Haoxiang Li, Haoyu Wu, Yangyang Gao, Liqun Zhang

**Affiliations:** 1 State Key Laboratory of Organic-Inorganic Composites, Beijing University of Chemical Technology, Beijing 100029, China; zhaoxy@mail.buct.edu.cn (X.Z.); spinebuct@163.com (Y.N.); 18810133881@163.com (H.L.); m18129227366@163.com (H.W.); zhanglq@mail.buct.edu.cn (L.Z.); 2 Key Laboratory of Beijing City on Preparation and Processing of Novel Polymer Materials, Beijing University of Chemical Technology, Beijing 100029, China; 3 Beijing Engineering Research Center of Advanced Elastomers, Beijing University of Chemical Technology, Beijing 100029, China

**Keywords:** percolated network, mixed nanoparticles, molecular dynamics simulation

## Abstract

The size of real nanoparticles (NPs) is polydisperse which can influence the electrical property of polymer nanocomposites (PNCs). Here, we explored the percolated network of mixed NPs with different sizes (small NPs and big NPs) by adopting a molecular dynamics simulation. The simulated results reveal that the big NPs are adverse to building the percolated network compared to the small NPs. Thus, the percolation threshold becomes higher along with increasing the mixing ratio, which denotes the concentration ratio of big NPs to the total NPs. For a better understanding of it, the dispersion state and the number and the size of clusters are employed to analyze the percolated network, which can explain the percolation threshold well. Furthermore, by adopting the Sun’s theory (Macromolecules, 2009, 42, 459–463), small and big NPs exhibit a weak antagonistic effect in the simulation if their total concentration is fixed. On the one hand, the number of small NPs is larger than that of big NPs at the same concentration. In addition, one big NP can connect to more others than one small NP. These two contrast effects are responsible for it. Interestingly, the shear flow leads to more contact aggregation structure of NPs which is beneficial to build the new percolated networks. Especially, the big NPs play a more important role in forming the percolated network than small NPs. Consequently, the percolation threshold is reduced at a higher shear rate. In total, our research work provides a further understanding of how the mixed NPs with different sizes form the percolated network in polymer matrix.

## 1. Introduction

Conductive polymer nanocomposites (PNCs) are consistent with the polymer matrix and conductive particles (NPs) (graphene, carbon nanotube (CNT), carbon black (CB)), which have been applied in many fields (for example the electromagnetic interference shielding, sensor and conductors) [1,2]. According to the percolation theory, the percolated network is built if the volume fraction of NPs is beyond a critical percolation threshold [3]. As a result, the conductivity rises rapidly which makes the materials change from an insulator to a conductor. The concentration, the dispersion state of the conductive NPs as well as their shape will influence the distribution state of NPs, which indirectly affect the percolated network. It is proved that the high concentration of NPs is very necessary to build the percolated network, but it will damage the flexibility, elasticity and processing properties, which thus is necessary to lower the percolation threshold.

Currently, there are lots of works on improving the electrical conductivity of PNCs by tuning various experimental parameters. For instance, the NP surface is modified to improve their miscibility with chains which can reduce the percolation threshold [4]. Meanwhile, due to the branched morphology of high-structure CBs, they are helpful to form the percolated network than low-structure ones [5]. When the surface of CNTs is covered with the small and highly conductive particles, the percolation threshold can be further lowered [6]. Moreover, a percolation model is developed to predict the percolation threshold by inserting the aspect ratio of CNTs [7]. Hybrid NPs with different shapes are usually introduced to achieve a high conductivity in the polycarbonate matrix [8]. Furthermore, the addition of the large spherical silica inside the polymer matrix leads to a strong percolated network of CBs, which reduces the percolation threshold [2]. In addition, it is an effective method to modify the NP surface by grafted chains, which can tune their spatial distribution and optimize the electrical conductivity [9,10]. The external fields will change the percolated network which impacts the conductivity. It shows that the tensile field induces the nonuniform distribution of NPs, which forms more new percolated paths [11]. Moreover, the electrical property of PNCs in the shear flow can be lower or higher than that in the quiescent state, which is related to the initial network structure [12,13]. Interestingly, the electrical conductivity is improved by several magnitudes over time in the quiescent conditions, which is attributed to the NPs reaggregation [14]. By utilizing the change of the percolation networks under the strain/stress or pressure condition, the environment conditions can be monitored [15]. However, the percolated network is difficult to characterize accurately in experimental studies. Computer simulation is an important method which is good at characterizing the percolated network. By adopting the molecular dynamics simulation, the percolation threshold changes in an opposite direction with the polymer-NP interaction [16]. This is due to the appearance of the local bridging NPs via the polymer chains at a high interaction. By adopting a Monte Carlo simulation, the relationship between the percolation threshold and the aspect ratio of fibers can be described by an exponential function [17]. The index parameters varies from 2.0 to 2.6 for two-dimensional networks and from 1.7 to 2.0 for three-dimensional networks [18,19]. The percolation threshold exhibits a continuous decline with increasing the NP stiffness in the stable state while it reaches a minimum value in the shear flow [18,20]. The uniform distribution state improves the distance between NPs, which is not beneficial to form the percolated network than a partial contact structure [21]. At similar sizes, the rod or plate NPs are much easier to build the percolated network with than the spherical NPs [22]. As for grafted NPs, the distribution state and the phase behavior are two factors impacting the percolation threshold which is proved by model and theory [23,24]. Moreover, the shear field will change the initial percolated network and then another network will be reformed [21]. This process is affected by the shape and size of NPs and the shear strength [21,25]. Finally, the percolated network will be destroyed if the shear strength is beyond the certain rate [26].

Carbon black (CB) has excellent sensitivity to small strain and low cost, which is widely applied as a conductive filler [27,28]. Currently, the spherical NPs are assumed to be monodisperse in terms of their size. However, the real NPs exhibit various sizes, which will change the percolated network and the conductive property. Meanwhile, mixed NPs with different sizes may produce the synergistic or antagonistic effect which still remains unclear to our knowledge. To answer it, a molecular dynamics simulation is employed to explore the conductive behavior of the mixed NPs (small NPs and big NPs) filled PNCs. Especially, the respective roles of small NPs and big NPs in constructing the percolated network are discussed which is beneficial to uncover the conductive mechanism of PNCs.

## 2. Models

In this work, by adopting a coarse-grained model, first the initial configuration of PNCs is constructed where each polymer is consistent of 30 beads [29]. In addition, each system contains 1600 polymer chains. The m and σ denote the mass and diameter in each polymer bead, respectively. The size distribution of NPs is set to be the bimodal distribution, which is consistent with the theoretical or experimental works [30,31]. Thus, two types of spherical NPs with different sizes are adopted in the matrix. Considering the computational power, the diameters of small and big NPs are fixed to 1σ and 4σ, respectively [11,32].

The modified Lennard–Jones interaction potential is employed to model the interactions between different beads [29], given by

(1) $$U_{ij}(r)=\left\{ \begin{array}{cc} 4\varepsilon_{ij}\left[{\left(\frac{\sigma }{r-r_{EV}}\right)}^{12}-{\left(\frac{\sigma }{r-r_{EV}}\right)}^{6}\right] & r-r_{EV}<r_{cutoff}\\ 0 & r-r_{EV}\geq r_{cutoff} \end{array}\right.$$

where $r_{cutoff}$ stands for the position where the interaction is truncated and shifted. The $r_{EV}$ denotes the excluded volume effect between the different beads. The interaction $\varepsilon_{\mathrm{ij}}$, the cutoff distance $r_{cutoff}$ and the $r_{EV}$ are presented in Table S1. These parameters can make the NPs relatively uniform distribution in the matrix, which can form the percolated network.

The chemical bonds within the polymer chains are simulated by the stiff finite extensible nonlinear elastic potential, given by

(2) $$V_{FENE}\;=\;-0.5kR_{0}^{2}ln[1-{\left(\frac{r}{R_{0}}\right)}^{2}]$$

where the *k* is set to be 30$\frac{\varepsilon }{\sigma^{2}}$ and $R_{0}$ is 1.5σ, which are consistent with the previous works [29].

Since we did not focus on a specific polymer chain, the reduced units are adopted for all physical quantities, such as the energy units ε, the length units σ, the mass units m, and the time unit τ($\tau ={\left(m\sigma^{2}/\varepsilon \right)}^{1/2}$). At the beginning, the initial configuration of PNCs is constructed with a lower density in a simulation box. The simulation systems are then equilibrated with the isothermal-isobaric (NPT) ensemble for 2 × 10^5^ τ with a timestep of δt = 0.001τ. The $P^{*}$ and $T^{*}$ are set to be 0.0 and 1.0, respectively, which are controlled by the Nose–Hoover barostat and thermostat. The periodic boundary condition is applied during the whole simulation. It is noted that the polymer chains have been checked to experience fully relaxed for each system. The simulation process for calculating the percolated network has been described in the previous works [16,33], which is also shown in the supporting information. The diagram of a typical polymer nanocomposite is presented in Figure S1, which can help to understand the simulated systems. Then, the 10,000 configurations are obtained to calculate the conductive probability whose time interval is chosen to be 10τ. The conductive probability is defined as the ratio of the number of the conductive configurations to that of all the configurations. Finally, the SLLOD methods are employed to simulate the shear flow, which is one widely used method for analyzing the shearing materials [34]. In addition, the special Lees–Edwards “sliding brick” boundary conditions [35] is adopted for the SLLOD equation. For more simulation details, refer to our previous works [16,33]. All MD runs are completed in the large scale atomic/molecular massively parallel simulator (LAMMPS) [36].

## 3. Results and Discussion

### 3.1. Ratio of Big Nanoparticles to the Total Nanoparticles

**Figure 1:**
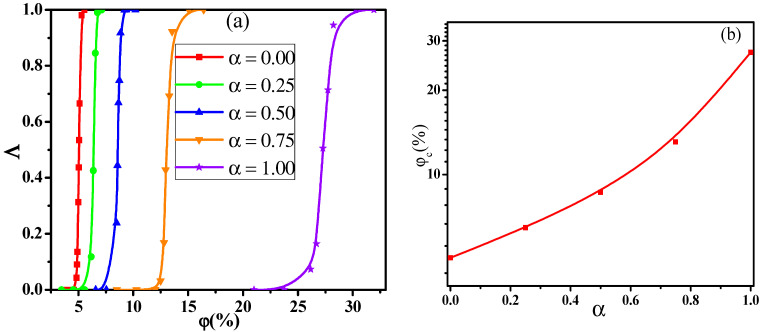
(**a**) Conductive probability Λ with the concentration φ of nanoparticles and (**b**) the percolation threshold $\varphi_{c}$ in respect of the mixing ratio (α).

In general, the NPs are available in a wide variety of sizes in the real system, which will in turn influence the conductive behavior of PNCs. Thus, to understand the conductive behavior, we first explored the dependence of the conductive probability Λ on the α. Here, the α denotes the mixing ratio of big NPs, which is calculated by the concentration ratio of big NPs to the total NPs. Figure 1a presents the change of the conductive probability Λ with the concentration (φ) of NPs for different mixing ratio α. The concentration of NPs is defined to be volume ratio of all the NPs to the simulation box. As the increase of the φ, the percolated network slowly grows up and becomes completed. Then, the formation of the percolated network quickly improves the Λ from 0 to 1. Here, the percolation threshold $\varphi_{c}$ is defined as the volume fraction of NPs at Λ = 0.5, which is calculated by fitting the curves in Figure 1a with a hyperbolic tangent equation: $\Lambda \;=\;0.5+0.5\tanh(2(\varphi -\varphi_{c})/d_{c})$ where *d_c_* denotes the percolation width [37,38]. As shown in Figure 1b, the $\varphi_{c}$ exhibits a monotonous rise with the increase of the α which reflects that small NPs can build the percolated network at a relatively low concentration. This is because the number of small NPs is much larger than that of big NPs at the same concentration, which helps to build the percolated network.

**Figure 2:**
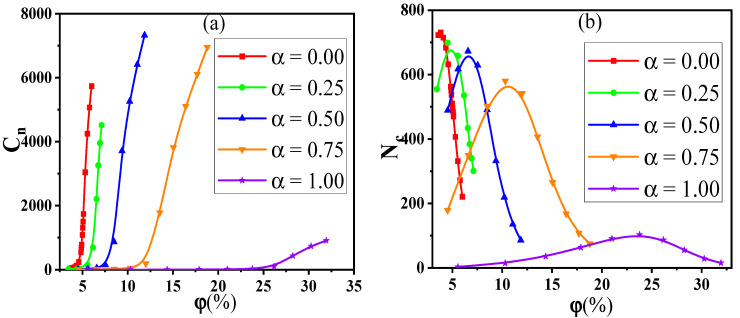
(**a**) The largest size $C_{n}$ and (**b**) the number $N_{c}$ of clusters with respect to the concentration φ of nanoparticles for various mixing ratios (α).

To further understand the results, the distribution state of NPs is analyzed by the radial distribution function (RDF). The peak position at r=1σ for α ≠ 1.0 or r = 4σ for α = 1.0 means the direct contact aggregation structure of NPs. The peak position at r=2σ for α≠ 1.0 or r = 5σ for α = 1.0 denotes the sandwiched structures of NPs. From Figure S2a, the RDF curves are similar for α< 1.0 due to more small NPs than big ones. Meanwhile, the low height of peaks in RDFs and the diagrams of NPs in Figure S2b can visually reflect a relatively uniform dispersion. The distribution behavior of particles can influence the conductive probability which however is more dependent on the percolated network. To analyze the percolated network, two important parameters are introduced, namely, the largest size $C_{n}$ and the number $N_{c}$ of clusters. The dependence of the $C_{n}$ and the $N_{c}$ on the φ for different α is presented in Figure 2. 

**Figure 3:**
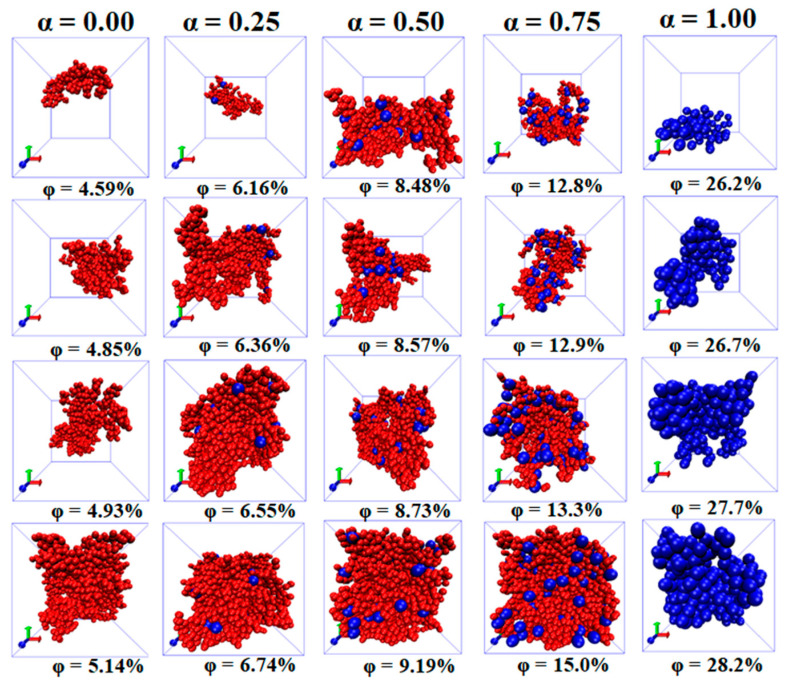
Diagrams of the largest cluster at four concentrations φ of nanoparticles (NPs) for different mixing ratios (α).

It is found that the $C_{n}$ rises slowly at first, and then, the rising speed becomes larger when the φ is beyond a critical value. Corresponding to it, the $N_{c}$ exhibits a nonmonotonic change, which reaches the maximum at the moderate φ. This indicates that the initial small clusters emerge to become large clusters with increasing the φ. Meanwhile, $N_{c}$ is reduced as α goes up because of the reduced number of NPs. Thus, the $C_{n}$ is more sensitive and accurate than the $N_{c}$ to represent the percolated network. Furthermore, the diagrams of the largest clusters are shown in Figure 3 for various α and φ, which reflects the formation process of percolated networks. The largest cluster is isolated at low φ and then becomes larger with the φ. At last, there appears a percolated network which leads to the high Λ. 

**Figure 4:**
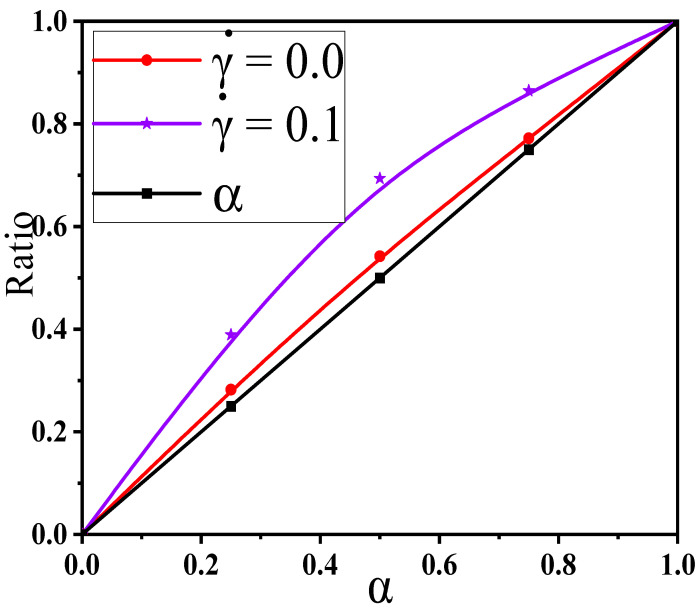
Ratio of big nanoparticles (NPs) to the whole NPs in the largest cluster for two shear rates $\dot{\gamma }$ for different mixing ratios (α).

To describe more in depth their respective roles in constructing the percolated network, the ratio of big NPs in the largest cluster to the total big NPs is calculated in respect of the mixing ratio α in Figure 4. 

Figure 4 also contains the concentration ratio α of big NPs to the whole NPs in the matrix (namely α), which presents a better comparison. It is found that this ratio (red line) rises with the α which however is a weakly higher than the α (black line). This means that big NPs take part more in constructing the percolated network than small ones. This is mainly due to the large size of big NPs which can connect more other NPs. However, this effect is very weak which is due to the uniform dispersion of NPs. Furthermore, according to the Sun’ theory [20], the percolation threshold can be calculated according to the excluded volume theory, given by

(3) $$\frac{\varphi^{SN}}{\varphi_{0}^{SN}}+\frac{\varphi^{BN}}{\varphi_{0}^{BN}}=1$$

where $\varphi^{SN}$ and $\varphi^{BN}$ are the respective concentrations of small and big NPs in the ternary systems when the system reaches the percolation state. Namely, the total concentration of NPs is the percolation threshold which is shown in Figure 1b. $\varphi_{0}^{SN}$ and $\varphi_{0}^{BN}$ are the percolation thresholds of one kind of NP, respectively. If the simulated result is below Sun’s line, it reflects the synergy. Conversely, it represents the antagonism.

**Figure 5:**
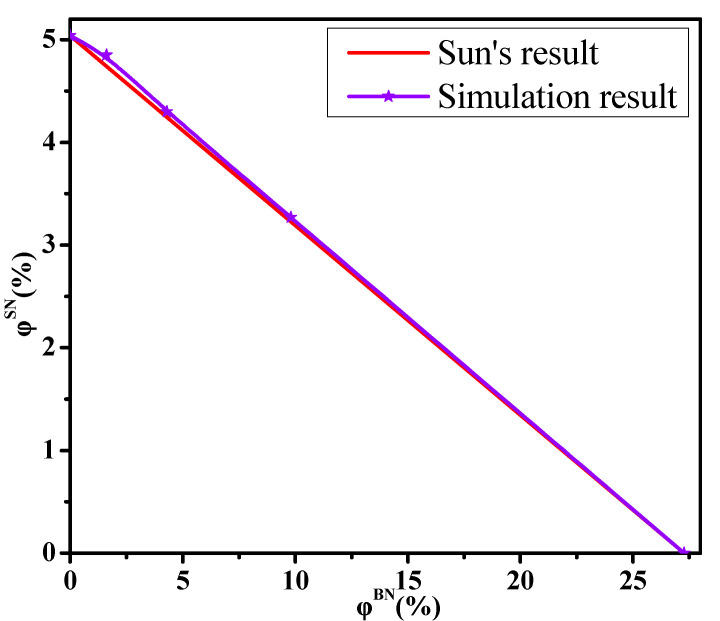
The dependence of the concentration of small nanoparticles (NPs) ($\varphi^{SN}$) on that of big NPs ($\varphi^{BN}$) at the percolation threshold.

**Figure 6:**
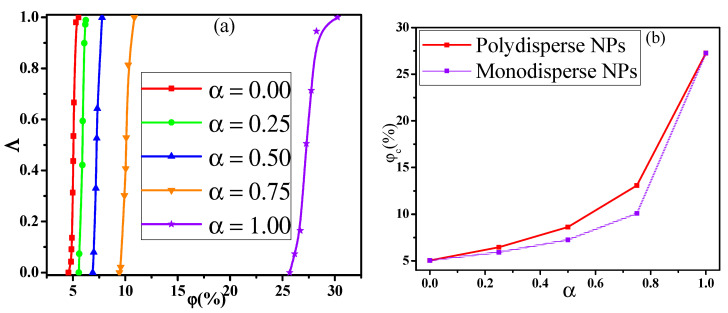
(**a**) Conductive probability Λ with the concentration φ of monodisperse nanoparticles and (**b**) their percolation threshold $\varphi_{c}$ in respect of the mixing ratio (α).

**Figure 7:**
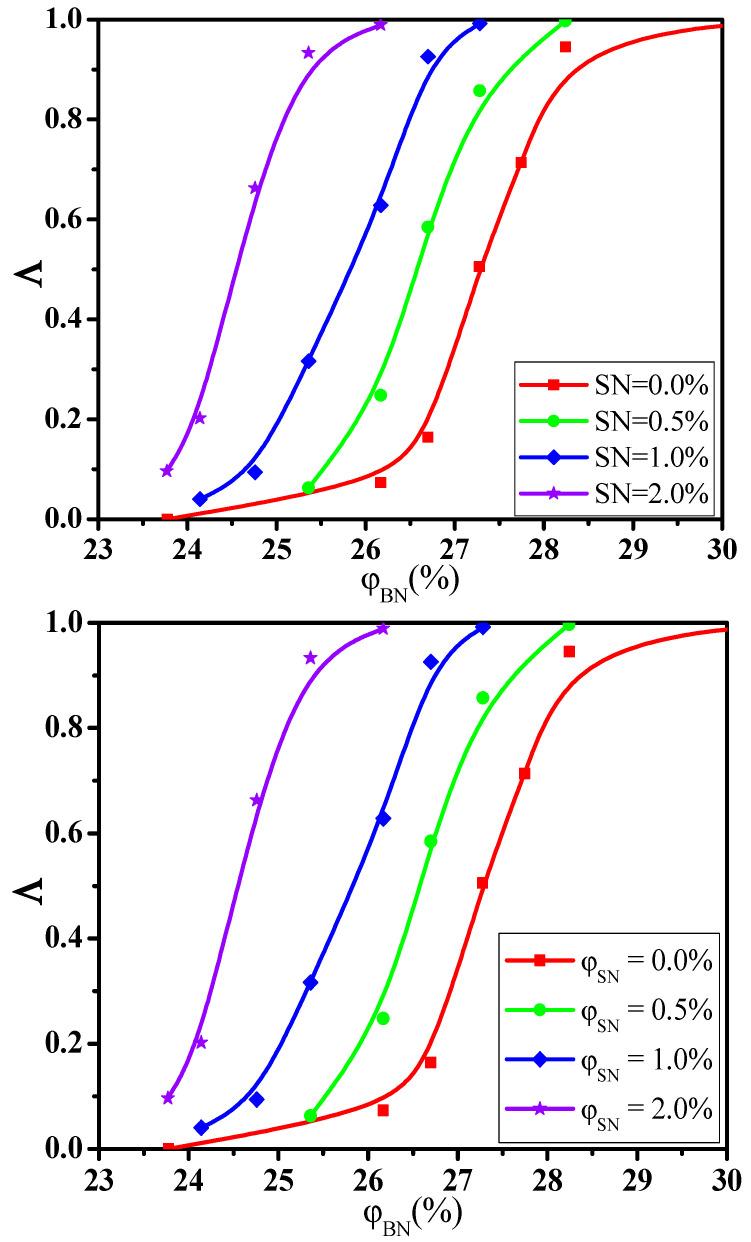
Conductive probability Λ with the concentration φ of big nanoparticles (BN) for three fixed concentrations of small nanoparticles (SN).

Figure 5 shows that the simulation values are slightly beyond that in Equation (3), which indicates a weak antagonistic effect. This is because on the one hand, the number of small NPs is larger than that of big NPs at the same concentration. On the other hand, one big NP can connect more other ones than one small NP. This can rationalize the obtained results. Furthermore, we analyzed the impact of the size polydispersity of NPs on the conductive probability Λ to describe more in depth their impact on building the percolated network. First, the corresponding averaged diameter (D_NP_) of NPs is calculated for different α in Figure S3. The D_NP_ is calculated as the following equation:

(4) $$D_{NP}{}^{3}(N_{1}+N_{2})=D_{1}{}^{3}N_{1}+D_{2}{}^{3}N_{2}$$

where $N_{1}$ and $D_{1}$ are the number and diameter of big NPs while $N_{2}$ and $D_{2}$ are the number and diameter of small NPs. The calculated Λ is presented in Figure 6a for the monodisperse NPs (namely, polydispersity index = 1.0). Similarly, the percolation threshold $\varphi_{c}$ is obtained which is shown in Figure 6b. It indicates that the $\varphi_{c}$ for monodisperse NPs is lower than that for polydisperse NPs. This indicates that the NPs with different sizes do not exhibit the synergistic effect which is consistent with the previous conclusion. However, it has reported a synergetic percolation for mixed fillers in experiments [39,40,41]. In these systems, the synergetic effect is realized by adding the additional second fillers into the first fillers which means that the total concentration of fillers increases. Figure 7 presents the change of the Λ with the $\varphi_{BN}$ at three fixed concentrations $\varphi_{SN}$ of small NPs as examples. The $\varphi_{c}$ is gradually reduced from the 27.3% to 24.5% with increasing the $\varphi_{SN}$, which proves the experimental results. In total, the percolation threshold gradually rises by increasing the mixing ratio of big NPs. However, small and big NPs exhibit a weak antagonistic effect if their total concentration is fixed.

### 3.2. Shear Field

**Figure 8:**
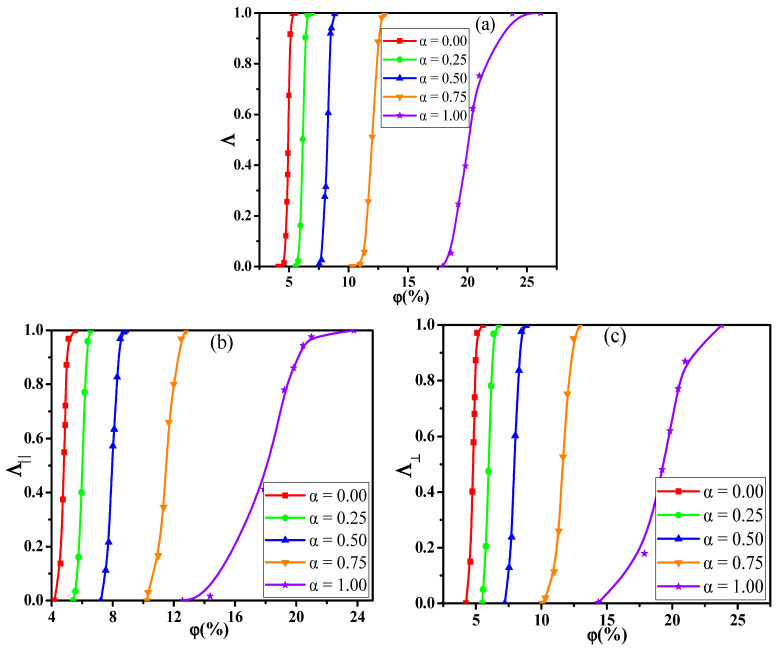
(**a**) Conductive probability Λ, (**b**) the directional $\Lambda_{∥}$ parallel to the shear direction, and (**c**) the directional $\Lambda_{⊥}$ perpendicular to the shear direction with the concentration φ of nanoparticles for different ratio (α).

**Figure 9:**
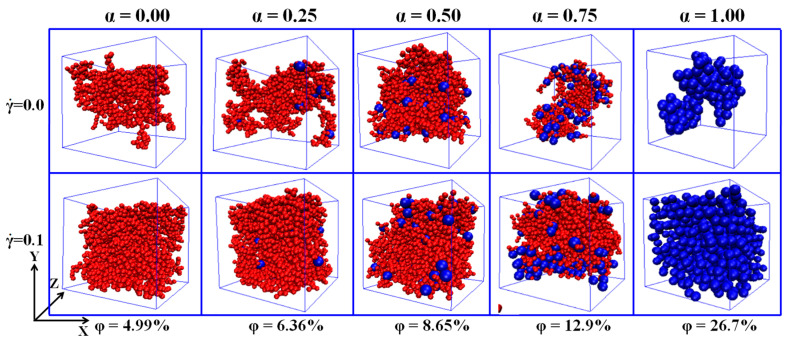
Snapshots of the largest clusters at two shear rates $\dot{\gamma \;}$ = 0.0 and 0.1 for different ratio (α).

**Figure 10:**
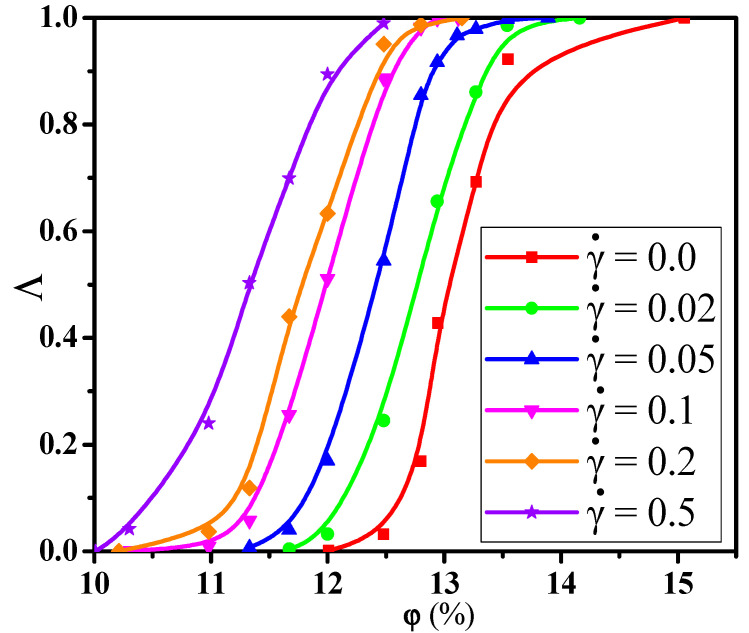
Conductive probability Λ with the concentration φ of nanoparticles for different shear rates ($\dot{\gamma }$).

**Figure 11:**
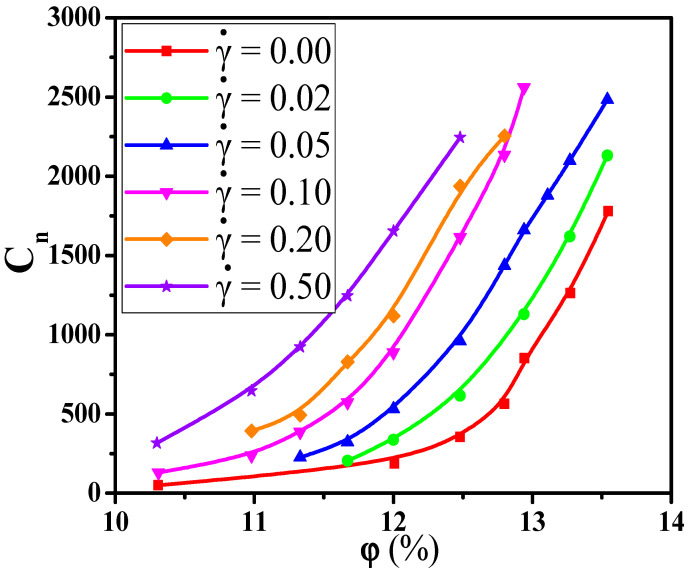
The largest cluster size $C_{n}$ with the concentration φ of nanoparticles for different shear rates ($\dot{\gamma }$).

In this section, we aimed to investigate the effect of the shear flow on the percolated network. First, Figure 8a shows the conductive probability Λ with the α for different φ by fixing the shear rate $\dot{\gamma }\;$= 0.1. The obtained percolation threshold $\varphi_{c}$ is put in Table S2 which slowly increases with increasing the α at $\dot{\gamma }\;$= 0.1. Meanwhile, the $\varphi_{c}$ at $\dot{\gamma \;}$= 0.1 is lower than that at $\dot{\gamma }\;$= 0.0. To understand it, Figure 8b,c presents the directional conductive probabilities $\Lambda_{∥}$ and $\Lambda_{⊥}$. The difference of $\varphi_{c}$ is small for $\Lambda_{∥}$ and $\Lambda_{⊥}$ in Figure S4. This is because the spherical NPs is isotropic along the shear direction. Then, we analyzed how the distribution state of NPs is changed by the shear flow where the α = 1.0 is chosen as an example in Figure S5. From the RDFs, the position at r = 4σ rises, reflecting that NPs appear more direct contact structures under the shear field. Meanwhile, one big NP can connect more other NPs than one small NP due to the size effect. Thus, both the direct contact aggregation structure and the size effect promote that big NPs participate more in forming the percolated network than small NPs. Namely, the concentration ratio of big NPs is higher at $\dot{\gamma \;}$= 0.1 than that at $\dot{\gamma \;}$= 0.0 which is presented in Figure 4. Furthermore, as shown in Figure S6, the largest cluster size $C_{n}$ gradually decreases with increasing the α which is in accord with $\varphi_{c}$. Some diagrams of the largest clusters are presented in Figure 9 at $\dot{\gamma }\;$= 0.0 and 0.1, which clearly presents the change of the percolated network. It is found that the $C_{n}$ is enhanced by the shear flow. In summary, more new percolated paths appear under the shear flow, which reduces the $\varphi_{c}$. At last, the α = 0.75 is taken as an example to clarify the relationship between the Λ and the $\dot{\gamma }$. The change of the Λ with the $\dot{\gamma }$ is calculated in Figure 10. The $\varphi_{c}$ is reduced from 13.0% to 11.3% with increasing the $\dot{\gamma }$ from 0.0 to 0.5. Then, both the $\Lambda_{∥}$ and $\Lambda_{⊥}$ are calculated in Figure S7 which gradually rise with increasing the $\dot{\gamma }$. This is attributed to more direct contact structures of NPs induced by the shear field. Furthermore, the $C_{n}$ is analyzed to characterize the percolated network in Figure 11, which is consistent with Λ. In total, the conductive property is improved with increasing the shear strength.

### 3.3. Discussion

When mapping this simulation systems to real polymers, the interaction energy ε is roughly 2.5–3.4 kJ·mol^−1^ for the common polymers [29]. The interfacial interaction is chosen to be 2.0 in this model, which means that the interaction energy is 5.0–6.8 kJ·mol^−1^. The average binding energy between polymer chains and silica particles is about 4.2–24 kJ·mol^−1^, which depends on the filler surface activity and chemical functionality [42,43]. Our simulated interfacial interaction is roughly within the experimental range. Meanwhile, the simulated persistence length is 0.68σ while it is 0.35–0.76 nm for real polymers [44]. Thus, the σ is roughly 1 nm. Furthermore, the diffusion coefficients (D) of NPs with different sizes are calculated which are 1.52 × 10^−2^$\tau^{-1}$ and 1.02 × 10^−3^$\tau^{-1}$, respectively. The simulated shear strength $\dot{\gamma }$ is 0.02–0.5$\tau^{-1}$ which is larger than the experimental values. Thus, the range of the Peclet number ($Pe=\frac{\gamma }{D}$) is from 1.3 to 490, which can be comparable to the experimental value [45]. Moreover, the dispersion states and network of NPs have also explored by this model [46,47,48,49], which are consistent with the theoretical and experimental results [50,51]. Thus, our models are relatively reasonable.

The conductive property of PNCs has been investigated, which is determined by the size, shape, volume fraction of NPs, the interfacial interaction and so on. For example, the dispersion state of rod NPs first changes from the aggregation to dispersion and then forms the local bridging structure. However, the percolation threshold shows an anti N-type with increasing the interfacial interaction [16]. However, the percolation threshold is reduced by increasing the interfacial interaction for spherical NPs [52]. The uniform distribution state improves the distances between NPs, which is no more beneficial to form the percolated network than a partial contact structure [21]. In addition, the dependence of the fiber aspect ratio on the percolation threshold follows the exponential function [17]. Especially, the index parameters vary from 2.0 to 2.6 for two-dimensional networks and from 1.7 to 2.0 for three-dimensional networks [18,19]. Moreover, the shape of NPs influences the formation of the percolated network, which reveals that the rod or plate NPs are more effective to improve the conductive probability than the spherical NPs [22]. The high stiffness of NPs can reduce the percolation threshold. However, it is minimum under the shear flow [18,20]. In this work, we mainly investigated the effect of mixed NPs with different sizes on the conductive probability of PNCs. It is found that there is a weak antagonistic effect for two kinds of NPs with different sizes at the fixed concentration of NPs. Meanwhile, the shear field is beneficial to reduce the percolation threshold compared with the quiescent state, which is attributed to the change of the percolated network. Moreover, in experiments, the effects of the polymer-NP interaction [4], the structures and aspect ratio of NPs [5,7], the chemical grafting modification of NPs [9,10] and the hybrid NPs with different shapes [8] on the conductive property of PNCs have been investigated. Moreover, the experiment reported that the percolation threshold of PNCs declines with the decrease of the filler size [53,54], which is consistent with our simulation result. However, the effect of mixed NPs on the formation of the percolated network has not been investigated in experiments to our knowledge. This maybe because the monodisperse NPs are difficult to obtain in experiments where NPs are normally polydisperse. Meanwhile, the size of NPs is difficult to be accurately determined. It is noted that some details of our model are not different with those of experimental reports, such as the size of polymer chains and NPs and the distribution state of NPs. Meanwhile, the conductive probability is used to denote the conductive property rather than the electrical conductivity. However, we are confident that some aspects of our model capture the behavior of the experimental systems. In particular, we simulated the interactions between polymer chains and NPs, which are within the experimental range. In addition, the size distribution of NPs in the model is consistent with that in experiments. Meanwhile, the simulated shear field is the same as that of the experiments. Lastly, how the surface size of NPs affects the formation of the percolated network is also an interesting topic, which deserves to be investigated in our further work.

## 4. Conclusions

In this work, a molecular dynamics simulation is employed to explore the formation of percolated networks of mixed nanoparticles (NPs) with different sizes (small NPs and big NPs) in polymer nanocomposites (PNCs). It is found that the big NPs are adverse for building the percolated network. As the increase of the concentration ratio of big NPs to the whole NPs, the percolation threshold rises from 5.04% to 27.3%. Furthermore, the percolated network is characterized by analyzing the dispersion state, the largest size and the number of clusters, which rationalizes the percolation threshold. Meanwhile, a weak antagonistic effect is observed for small and big NPs at the fixed concentration of NPs. On one hand, the number of small NPs is larger than that of big NPs at the same concentration. Meanwhile, one big NP can connect more other NPs than one small NP. These two contrast effects can explain the results. Furthermore, the shear field is beneficial to improve the conductive probability compared with the quiescent state. This is because the NPs form more direct contact aggregation structures which can link other particles to build the new percolated network. Therefore, the big NPs participate more in forming the new percolated paths than small NPs. Finally, the percolation threshold is lowered with increasing the shear strength. In total, our work provides further understanding of the effect of the mixed NPs with different sizes on building the percolation network of PNCs.

## Data Availability

Data sharing is not applicable to this article.

## Supplementary Materials

The following are available online at https://www.mdpi.com/article/10.3390/ma14123301/s1, Figure S1. (a) The radial distribution function for different mixing ratios (α) where the concentration φ of nanoparticles (NPs) is their percolation threshold. (b) Diagrams of NPs where the polymer chains are neglected for clarity, Figure S2. The averaged diameter (D_NP_) of nanoparticles for different mixing ratios α, Figure S3. The percolation threshold $\varphi_{c}$ for the parallel directional conductive probability $\Lambda_{∥}$ and perpendicular directional conductive probability $\Lambda_{⊥}$ in respect of the mixing ratio α, Figure S4. The radial distribution function of nanoparticles for the mixing ratio α = 1.0, Figure S5. The largest size $C_{n}$ of cluster as a function of the concentration φ of nanoparticles for different mixing ratios (α), Figure S6. (a) The parallelly directional conductive probability $\Lambda_{∥}$ and (b) perpendicularly directional conductive probability $\Lambda_{⊥}$ as a function of the concentration φ of nanoparticles for different shear rates ($\dot{\gamma }$), Table S1 All the interaction parameter $\varepsilon_{\mathrm{ij}}$, the cutoff distance $r_{cutoff}$ and the $r_{EV}$.
