# Supplementary material for: Percolated Network of Mixed Nanoparticles with Different Sizes in Polymer Nanocomposites: A Coarse-Grained Molecular Dynamics Simulation
Source: Materials (Basel). 2021 Jun 15;14(12):3301. doi: 10.3390/ma14123301 (PMC8232767; doi:10.3390/ma14123301)
Supplement: Supplementary file 1 [file materials-14-03301-s001.zip › materials-1239890-SI.pdf]

materials

Supplementary Materials

Percolated Network of Mixed Nanoparticles with Different Sizes in Polymer Nanocomposites: A Coarse-Grained Molecular Dynamics Simulation

Xiuying Zhao [1,2,3], Yun Nie [1,2,3], Haoxiang Li [1,2,3], Haoyu Wu [1,2,3], Yangyang Gao [1,2,3,*] and Liqun Zhang [1,2,3]

1 State Key Laboratory of Organic-Inorganic Composites, Beijing University of Chemical Technology, Beijing 100029, China; zhaoxy@mail.buct.edu.cn (X.Z.); spinebuct@163.com (Y.N.); 18810133881@163.com (H.L.); m18129227366@163.com (H.W.); zhanglq@mail.buct.edu.cn (L.Z.)
2 Key Laboratory of Beijing City on Preparation and Processing of Novel Polymer Materials, Beijing University of Chemical Technology, Beijing 100029, China
3 Beijing Engineering Research Center of Advanced Elastomers, Beijing University of Chemical Technology, Beijing 100029, China
* Correspondence: gaoyy@mail.buct.edu.cn

Citation: Zhao, X.; Nie, Y.; Li, H.; Wu, H.; Gao, Y.; Zhang, L. Percolated Network of Mixed Nanoparticles with Different Sizes in Polymer Nanocomposites: A Coarse-Grained Molecular Dynamics Simulation. *Materials* **2021**, *14*, 3301. https://doi.org/10.3390/ma14123301

Academic Editor(s): Andrzej Dziedzic

Received: 13 May 2021
Accepted: 8 June 2021
Published: 15 June 2021

Publisher's Note: MDPI stays neutral with regard to jurisdictional claims in published maps and institutional affiliations.

Copyright: © 2021 by the authors. Licensee MDPI, Basel, Switzerland. This article is an open access article distributed under the terms and conditions of the Creative Commons Attribution (CC BY) license (http://creativecommons.org/licenses/by/4.0/).

The simulation process for calculating the percolated network

In order to characterize the percolated network, the tunneling distance (TD) is introduced to judge whether any two NPs are connected. This is because the electrons are considered to be transferring between NPs via quantum tunneling. TD reflects the contact conductance between a pair of fillers which can not be estimated accurately either theoretically or experimentally [1]. Here, the TD is chosen to be 1.0σ in this work.[2] The TD affects the absolute conductivity, but does not influence the relativity. At the first step, a site number and a cluster number are assigned for each NP. The site number is equal to the cluster number, ranging from 1 to N, where N is the total number of NPs. Then, each NP is checked for connection with others. If two NPs are connected, they will be assigned a common cluster number. Finally, all the NPs with the same cluster number are in the same cluster while NPs with different cluster numbers are not connected. If the NP network spans one direction continuously from one side to another, the system is conductive in this direction. If the NP network spans the three-dimensional directions continuously, the system is homogeneously conductive in three directions.

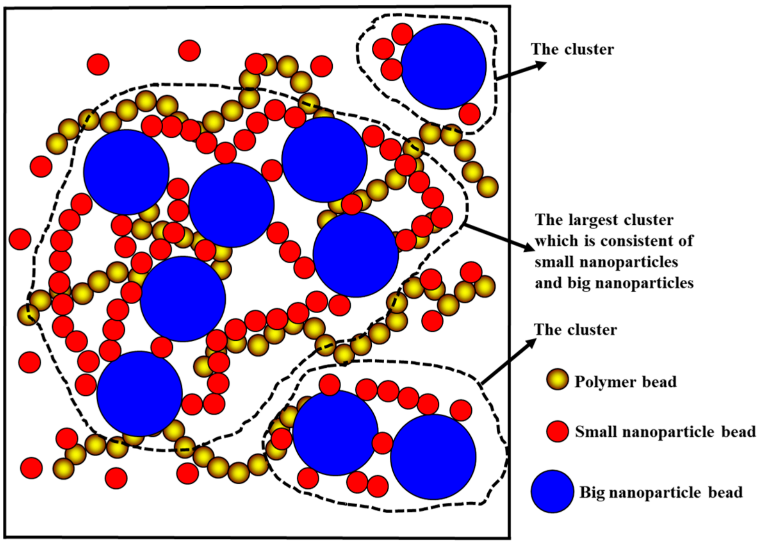

Figure S1. The diagram of a typical polymer nanocomposite which contains polymer, small nanoparticles and big nanoparticles. The yellow beads denote the polymer beads, the red beads denote

the small nanoparticles and the blue beads denote the big nanoparticles. Meanwhile, some clusters are presented which are determined by the distances between nanoparticles.

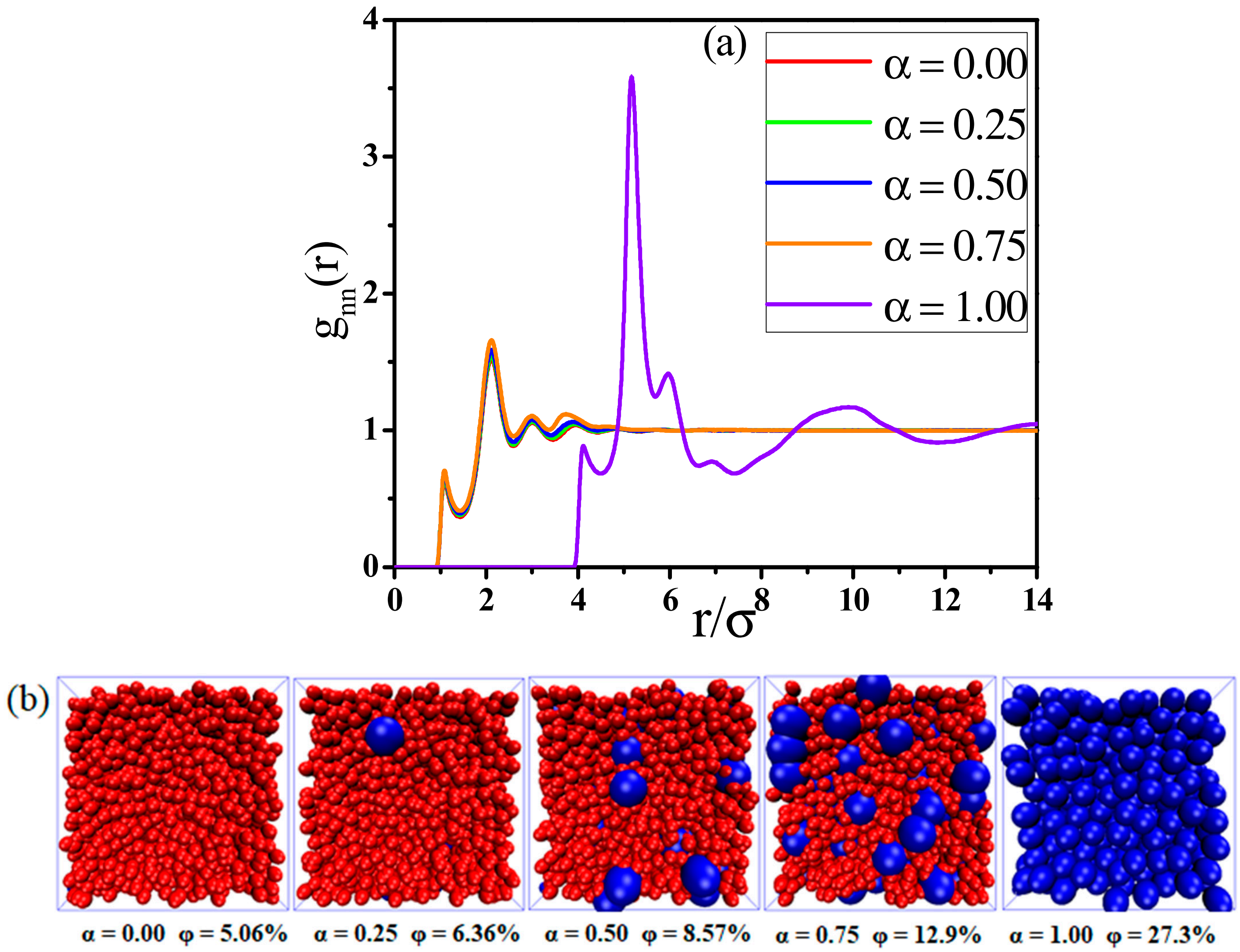

Figure S2. (**a**) The radial distribution function for different mixing ratios (α) where the concentration φ of nanoparticles (NPs) is their percolation threshold. (**b**) Diagrams of NPs where the polymer chains are neglected for clarity.

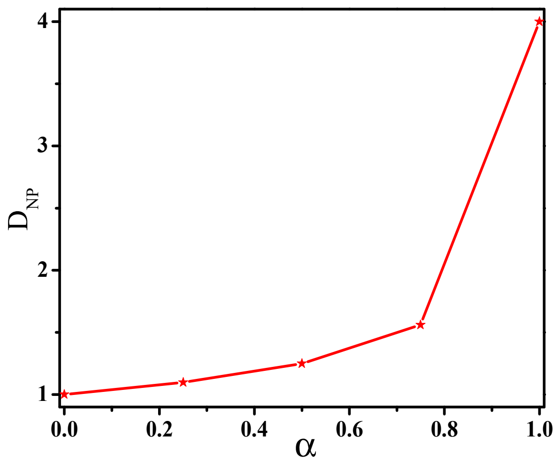

Figure S3. The averaged diameter (D_{NP}) of nanoparticles for different mixing ratios α.

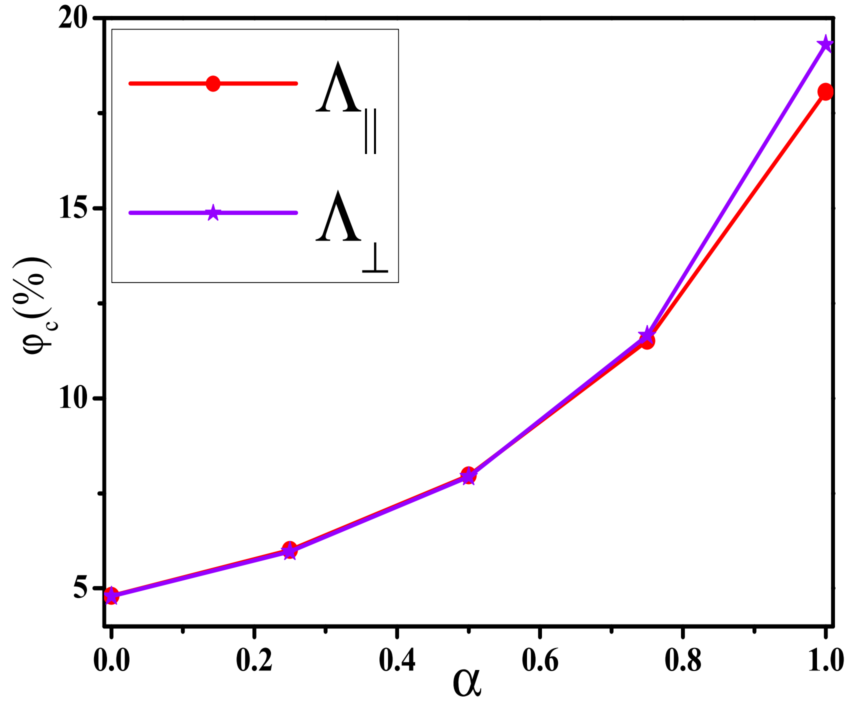

Figure S4. The percolation threshold φ_c for the parallel directional conductive probability $\Lambda_{\|}$ and perpendicular directional conductive probability $\Lambda_{\perp}$ in respect of the mixing ratio α .

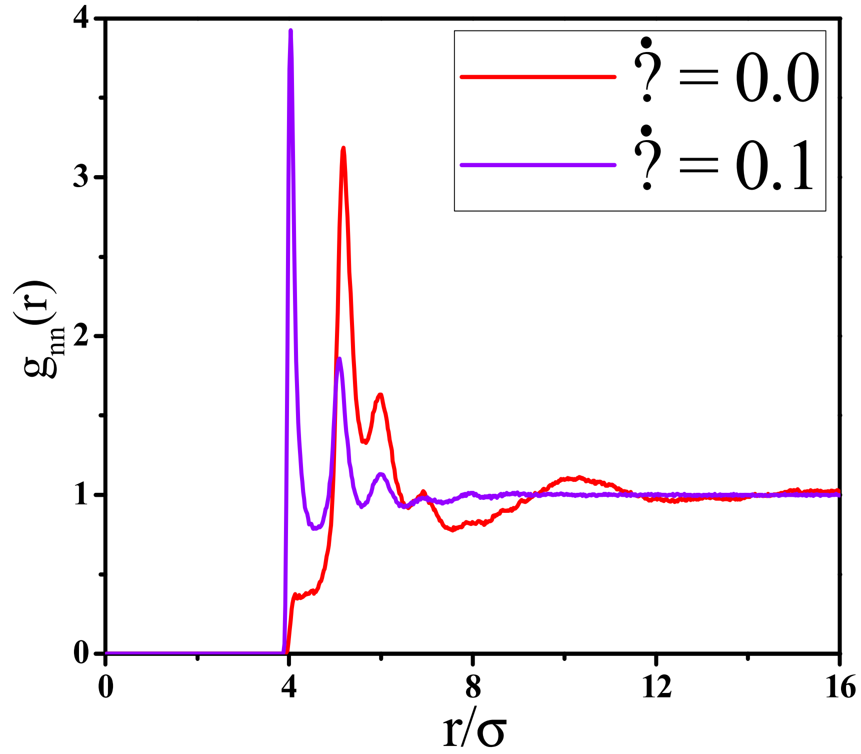

Figure S5. The radial distribution function of nanoparticles for the mixing ratio α =1.0.

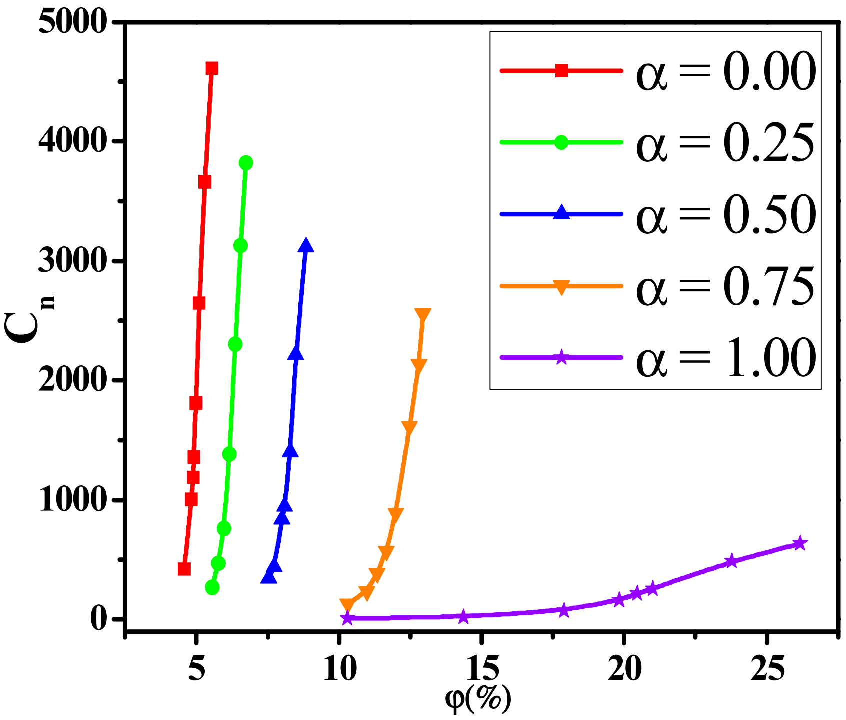

Figure S6. The largest size C_n of cluster as a function of the concentration φ of nanoparticles for different mixing ratios (α).

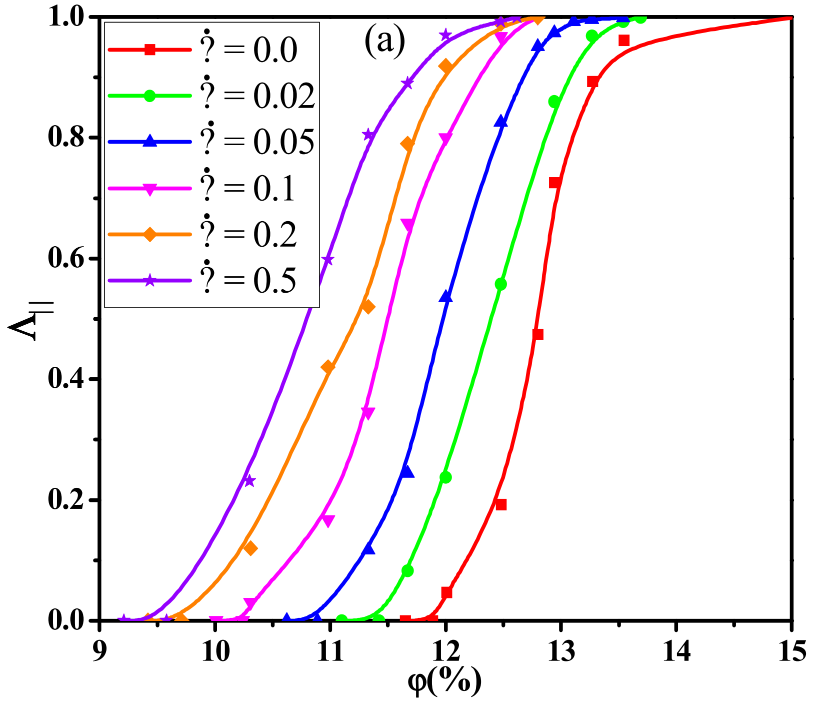

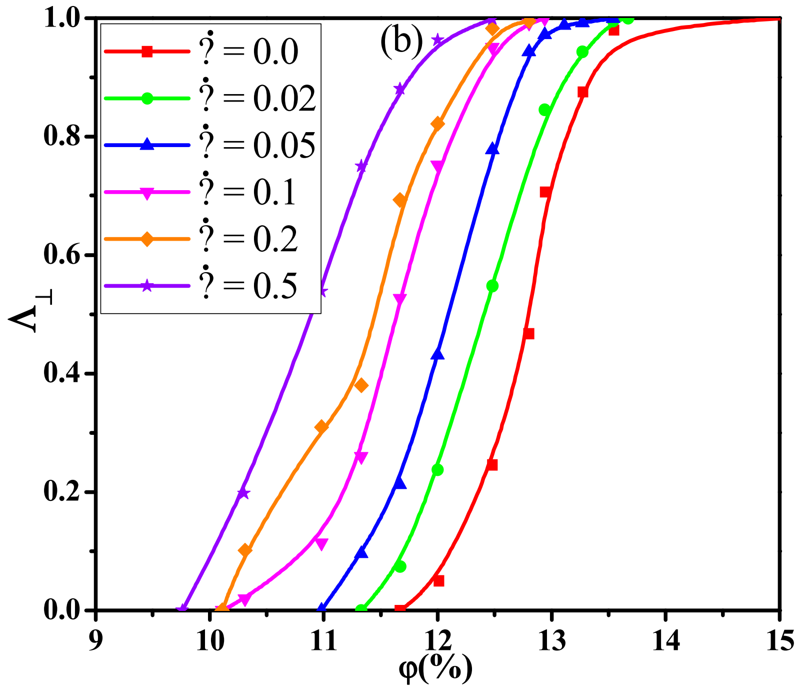

Figure S7. (**a**) The parallelly directional conductive probability $\Lambda_{\parallel}$ and (**b**) perpendicularly directional conductive probability $\Lambda_{\perp}$ as a function of the concentration φ of nanoparticles for different shear rates ($\dot{\gamma}$).

Table S1. All the interaction parameter ε_{ij}, the cutoff distance r_{cutoff} and the r_{EV} .

Type	ε_{ij}	r_{cutoff}	r_{EV}
Polymer-polymer	1.0	2.5	0
Polymer-small NPs	2.0	2.5	0
Polymer-big NPs	2.0	2.5	1.5
Small NPs-big NPs	1.0	1.12	1.5
Small NPs-big NPs	1.0	1.12	1.5
Big NPs-big NPs	1.0	1.12	3.0

Table S2. The percolation threshold φ_c at two shear rates $\dot{\gamma}$ in respect of the mixing ratio (α).

Systems	Ratio (α)	φ_c ($\dot{\gamma}$=0.0)	φ_c ($\dot{\gamma}$=1.0)
1	0.00	5.04	4.96
2	0.25	6.46	6.16
3	0.50	8.63	8.20
4	0.75	13.1	12.0
5	1.00	27.3	20.1

References

1. Wang Y, Weng GJ, Meguid SA, Hamouda AM. A continuum model with a percolation threshold and tunneling-assisted interfacial conductivity for carbon nanotube-based nanocomposites. *J. App. Phys*. **2014**, *115*, 193706.
2. Zhang H, Li H, Hu F, Wang W, Zhao X, Gao Y, et al. Cavitation, crazing and bond scission in chemically cross-linked polymer nanocomposites. *Soft Matter*. **2019**, *15*, 9195–9204.
